# Increased Frontal Response May Underlie Decreased Tinnitus Severity

**DOI:** 10.1371/journal.pone.0144419

**Published:** 2015-12-14

**Authors:** Jake R. Carpenter-Thompson, Sara Schmidt, Edward McAuley, Fatima T. Husain

**Affiliations:** 1 Neuroscience Program, University of Illinois Urbana-Champaign, Champaign, Illinois, United States of America; 2 Medical Scholars Program, University of Illinois Urbana-Champaign, Champaign, Illinois, United States of America; 3 Department of Speech and Hearing Science, University of Illinois Urbana-Champaign, Champaign, Illinois, United States of America; 4 Beckman Institute, University of Illinois Urbana-Champaign, Champaign, Illinois, United States of America; 5 Department of Kinesiology and Community Health, University of Illinois Urbana-Champaign, Champaign, Illinois, United States of America; University of California, San Francisco, UNITED STATES

## Abstract

**Objectives:**

The overall goal of the study was to identify functional and behavioral differences between individuals with higher tinnitus distress and individuals with lower tinnitus distress. Subsequent exploratory analyses were conducted to investigate the role physical activity may have on the observed results between high and low distress groups. The purpose of the experiment was to identify brain regions to be targeted in future intervention studies for tinnitus.

**Design:**

A total of 32 individuals with varying levels of tinnitus severity were recruited from the Urbana-Champaign area. Volunteers were divided into higher tinnitus distress (HD) and lower tinnitus distress (LD) groups. Note that these groups also significantly differed based on physical activity level and were subsequently stratified into higher and lower physical activity level subgroups for exploratory analysis. While in a functional magnetic resonance imaging (fMRI) scanner, subjects listened to affective sounds classified as pleasant, neutral or unpleasant from the International Affective Digital Sounds database.

**Results:**

The HD group recruited amygdala and parahippocampus to a greater extent than the LD group when listening to affective sounds. The LD group engaged frontal regions to a greater extent when listening to the affective stimuli compared to the HD group. Both higher physical activity level subgroups recruited more frontal regions, and both lower levels of physical activity subgroups recruited more limbic regions respectively.

**Conclusion:**

Individuals with lower tinnitus distress may utilize frontal regions to better control their emotional response to affective sounds. Our analysis also suggests physical activity may contribute to lower tinnitus severity and greater engagement of the frontal cortices. We suggest that future intervention studies focus on changes in the function of limbic and frontal regions when evaluating the efficacy of treatment. Additionally, we recommend further investigation concerning the impact of physical activity level on tinnitus distress.

## Introduction

The population of older adults is rapidly increasing and individuals in this group are living longer. Despite living longer, a commonly accepted aspect of normal aging is a progressive alteration and decline in quality of life (QOL) [1]. For some, this reduced QOL may be associated with tinnitus, ringing in the ears, which affects upwards of 33% of adults age 65 years and older [2]. Previous research has suggested the tinnitus signal is similar to an alarm bell, and the limbic system is responsible for attributing emotional significance to the percept, resulting in intrusive and chronic tinnitus [3, 4]. Tinnitus may result in loss of sleep, interference with concentration, social withdrawal, avoidance behaviors, and negative emotional reactions [5–8]. Past studies have suggested that depression and anxiety may be strongly correlated with tinnitus severity [6, 7]. A lack of effective coping strategies may lead to the development of distress factors and a decreased QOL. Higher levels of physical activity have been associated with lower levels of tinnitus severity [9]. However, the neuronal differences underlying this association have not been investigated. The primary aim of the current study was to utilize functional magnetic resonance imaging (fMRI) to characterize neuronal differences among tinnitus subjects with higher tinnitus distress (HD) and individuals with lower tinnitus distress (LD). An exploratory aim was to analyze subgroups within the HD and LD groups, stratified based on physical activity level, to identify any beneficial effects that may be associated with higher physical activity levels in the tinnitus population. This project serves as a baseline to identify brain regions to be targeted in a future intervention study that directly tests the efficacy of novel therapies for tinnitus, such as physical activity.

Alterations in the limbic system have been associated with tinnitus distress. Key regions of the limbic system associated with tinnitus distress include the amygdala, parahippocampus, and insula [10–14]. The amygdala was one of the first limbic regions to be associated with tinnitus distress [3, 11, 15]. Previous research has suggested that the amygdala may attribute emotional significance to sounds, thus determining the individual’s emotional response to stimuli [16, 17]. Similarly, studies have suggested that increased parahippocampal and insular activation in tinnitus groups compared to controls may also be neural indicators of tinnitus related distress [10, 12, 13]. Previous research in our lab has demonstrated differences in emotional processing between groups of individuals with slight differences in levels of tinnitus distress using an affective sound categorization task and fMRI [14]. Note that these groups ranged from slight to mild tinnitus distress and primarily differed in the period of time they had tinnitus [14]. We expected these differences to be more evident when comparing tinnitus groups with larger differences in tinnitus distress. Therefore, we expected increased response in the discussed limbic regions in the HD group compared to the LD group.

In accordance with past research that demonstrated increased frontal response in individuals with lower tinnitus severity levels [13], we expected the LD group would recruit frontal regions to a greater extent than the HD group. Increased response from frontal regions when processing emotionally salient stimuli has been associated with improved control over emotional response [18, 19]. Therefore, we hypothesized the LD group would have better emotional control resulting in lower tinnitus severity, and this would be reflected in increased frontal response when compared to the HD group. We also suspected that these alterations in response patterns may be related to physical activity. Our recent survey study [9] aimed to examine the relationship between physical activity and tinnitus severity. Data from 1030 individuals with tinnitus, with a completion rate of approximately 60%, were collected, which included measures of tinnitus severity, physical activity, and quality of life. Increased levels of physical activity were found to be significantly correlated with lower tinnitus severity and increased quality of life in all statistical analyses. Additionally, tinnitus distress was strongly correlated with the mental component score, a representation of anxiety and depression. Based on previous studies demonstrating an association between physical activity level and tinnitus severity [9] and others demonstrating an association between physical activity, improved quality of life, decreased anxiety and depression [1, 20–22] and increased frontal response [21], we expected physical activity to contribute to enhanced emotional regulation of the response to tinnitus.

There is a gap in knowledge concerning functional differences between individuals with LD and HD. It is important to investigate these differences to identify areas that may be hubs of tinnitus distress or successful coping for future intervention studies. Therefore, the aim of the present study was to identify functional and behavioral differences between HD and LD groups to suggest brain regions to use as targets in a future intervention studies for tinnitus. An exploratory aim, based on previous survey research [9] suggesting an association between physical activity and tinnitus distress, was to investigate how physical activity may contribute to the observed results. Our main hypotheses were as follows 1. The HD group would exhibit increased response in the amygdala, parahippocampus and insula compared to the LD group, and 2. The LD group would show elevated response in frontal regions compared to the HD group. We also speculated that physical activity level may contribute to our main hypotheses, and more specifically, that higher levels of physical activity may be associated with increased response in frontal regions.

## Methods

### Subjects

Subjects were recruited from the Urbana-Champaign area using emails and flyers. This research was approved by the University of Illinois at Urbana-Champaign Institutional Review Board (IRB), and prior to participation, subjects provided written informed consent in accordance with the IRB (University of Illinois Urbana-Champaign Institutional Review Board protocol 12896). Adults age 30–70 with tinnitus free of neurological and psychological disorders, surgical implants, prior head trauma, hyperacusis, and profound hearing loss were included in the study. Monetary compensation was provided to the subjects upon study completion. Subjects were grouped based on their tinnitus severity scores. Subjects scoring ≤18 on the Tinnitus Handicap Inventory (THI) (slight-mild) were included in the low tinnitus distress group (LD), and subjects scoring ≥20 on the THI scale (mild-moderate) were included in the high tinnitus distress group (HD) (Table 1). Note that subjects in the LD group also had significantly higher physical activity scores than those in the HD group. An exploratory analysis was conducted to better isolate the effects of physical activity on severity. The LD and HD groups were divided into subgroups based on median Godin Leisure Time Exercise Questionnaire (GLTEQ) scores for the exploratory within group analyses. For the within group analyses, the LD group was divided into a higher physical activity level subgroup (LD_HPS) and a lower physical activity level subgroup (LD_LPS). Additionally, the HD group was divided into a higher physical activity level subgroup (HD_HPS) and a lower physical activity level subgroup (HD_LPS) (Table 2).

**Table 1:** Demographic and clinical characteristics for both groups.

	LD	HD
**Group Size**	16	16
**Gender**	13 Male 3 Female	7 Male 9 Female
**Age (M±SD)**	49.3 ± 11.5	53.9 ± 9.5
**THI (M±SD)**	9.6 ± 5.7*	34.5 ± 9.7*
**TFI (M±SD)**	14.9 ± 7.6*	46.1 ± 12.1*
**GLTEQ (M±SD)**	57.7 ± 30.2*	27.3 ± 17.9*
**BAI (M±SD)**	1.5 ± 2.1*	4.9 ± 3.4*
**BDI-II (M±SD)**	3.3 ± 4.6	7.3 ± 7.5

LD = Lower Tinnitus Distress, HD = Higher Tinnitus Distress, THI = tinnitus handicap inventory, TFI = Tinnitus Functional Index, GLTEQ = Godin leisure time exercise questionnaire, BAI = Beck anxiety inventory, BDI-II = Beck depression inventory. Age, THI, TFI, GLTEQ, BAI and BDI-II scores were compared using independent sample *t*-tests.

* Indicates significant difference at p<0.01.

**Table 2:** Demographic and clinical characteristics for the within group split of the LD (left two columns) and HD (right two columns) groups based on median GTLEQ scores.

	Low Distress Subgroups		High Distress Subgroups	
	LD_HPS	LD_LPS	HD_HPS	HD_LPS
**Group Size**	8	8	8	8
**Gender**	7 Male 1 Female	6 Male 2 Female	5 Male 3 Female	2 Male 6 Female
**Age (M±SD)**	53.5 ± 9.9	54.4 ± 9.7	45.4 ± 13.8	53 ± 7.7
**THI (M±SD)**	9.0 ± 5.9	10.3 ± 5.8	34.3 ± 9.9	34.8 ± 10.4
**TFI (M±SD)**	16.0 ± 9.7	13.9 ± 5.2	44.7 ± 13.7	47.6 ± 10.9
**GLTEQ (M±SD)**	80.6 ± 24.8 *	34.6 ± 11.2*	40.5 ± 14.7*	14.1 ± 8.7*
**BAI (M±SD)**	0.3 ± 0.7	2.75 ± 2.4	3.3 ± 1.9	6.6 ± 3.7
**BDI-II (M±SD)**	1.0 ± 2.1	5.6 ± 5.4	6.4 ± 7.4	8.1 ± 8.0

LD = Lower Tinnitus Distress, HD = Higher Tinnitus Distress, HPS = Higher Physical Activity Subgroup, LPS = Lower Physical Activity Subgroup, THI = Tinnitus Handicap Inventory, TFI = Tinnitus Functional Index, GLTEQ = Godin leisure time exercise questionnaire, BAI = Beck anxiety inventory, BDI-II = Beck depression inventory.

* Indicates significant difference at p<0.01.

### Measures

Tinnitus severity was measured using the tinnitus functional index (TFI) and tinnitus handicap inventory (THI) [9, 13, 23–26]. The TFI provides an overall score (0–100) of tinnitus severity summed from the impact of tinnitus on 8 domains [25]. The THI asks questions pertaining to the impact of tinnitus on an individual’s lifestyle and general well-being to provide a comprehensive score between 0 and 100 and provides categories of distress (0–16 slight, 18–36 mild, 38–56 moderate, 58–76 severe, 78–100 catastrophic) [24]. The TFI and THI are questionnaires commonly used to estimate tinnitus severity [9, 13, 23–26].

Anxiety and depression were assessed using the Beck anxiety inventory (BAI) and Beck depression inventory (BDI-II) respectively [27–30]. The BAI includes 21 targeted questions to provide a score ranging from 0–63 (0–7 minimal, 8–15 mild, 16–25 moderate, 26–63 severe) [31, 32]. The BDI-II also consists of 21 questions with scores ranging from 0–63 (0–9 minimal, 10–18 mild, 19–29 moderate, 30–63 severe) [29]. The BAI and BDI-II have been validated as measures of anxiety and depression respectively [27–30].

Physical activity level was assessed using the Godin leisure time exercise questionnaire (GLTEQ) [33]. Subjects were asked how many times during a typical week they participated in strenuous (e.g. running, jogging, basketball, football), moderate (e.g., fast walking, tennis, dancing, badminton) and mild (e.g. archery, fishing, bowling, golf) forms of activity. Their responses were multiplied by their respective metabolic equivalents (9, 5, and 3 for strenuous, moderate, and mild) to obtain a total physical activity score [33]. In the present study, GTLEQ scores were used to compare physical activity level between groups in order to determine relative higher and lower physical activity scores. The GTLEQ has been used in several studies to assess physical activity [9, 33–38].

### Audiological assessment

**Fig 1:**
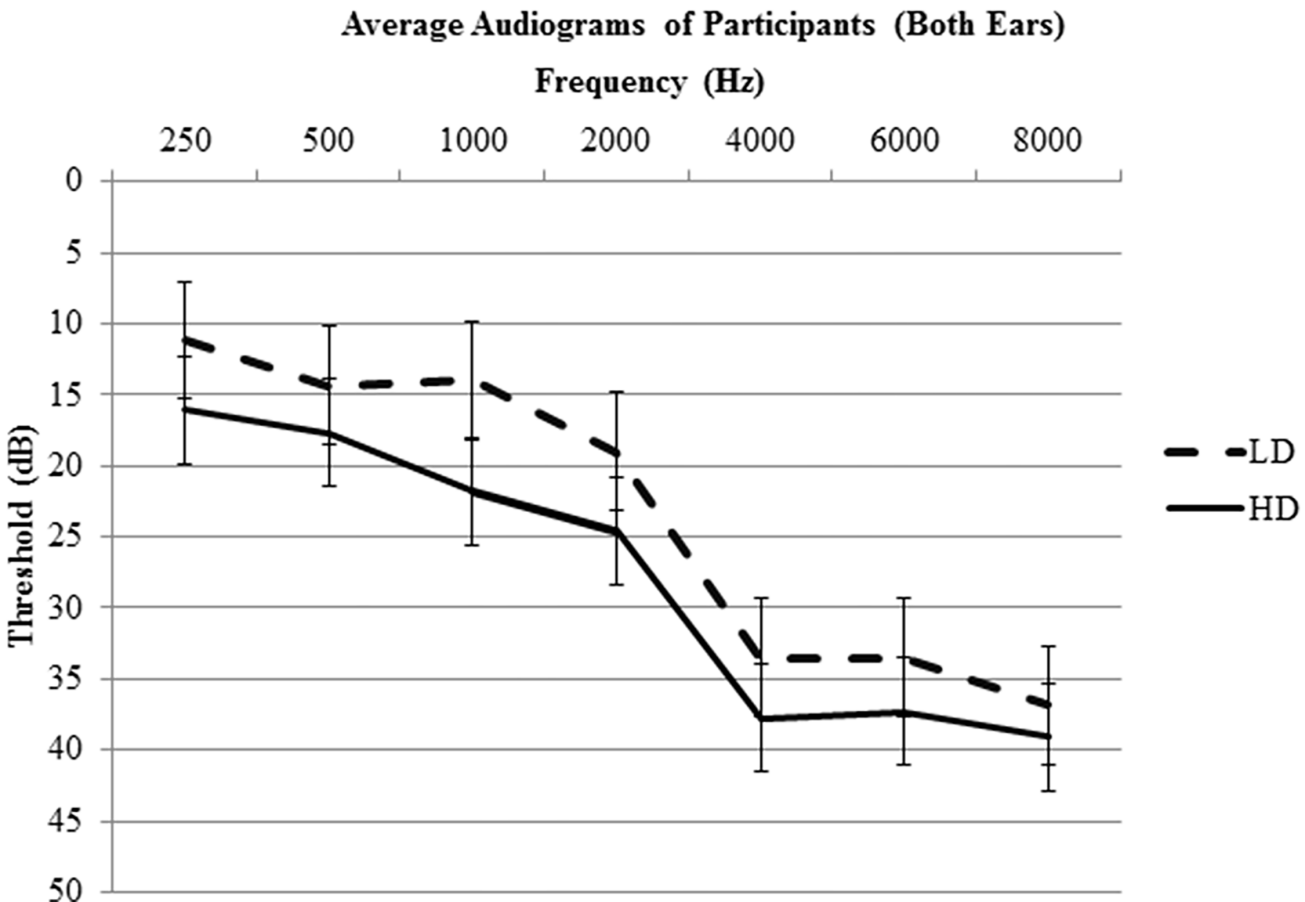
Average audiograms (combined values of both ears) with standard error bars. No significant differences between groups at the tested frequencies were observed (p<0.05).

A complete audiological assessment was performed on each subject. The audiological assessment included pure tone audiometry, word recognition, bone conduction, and tympanometry testing. Pure tone thresholds for the following frequencies were tested: 250, 500, 1000, 2000, 4000, 6000, and 8000 Hz. The right and left ear pure tone thresholds were averaged to provide an overall hearing profile (Fig 1). The averaged pure tone thresholds ranged from normal hearing to moderate hearing loss (0–40 dB HL) for both groups (Fig 1). Note that upon direct comparison between groups, hearing thresholds did not significantly differ (p<0.05). One subject was excluded for asymmetrical profound hearing loss (90+ dB HL) at all tested frequencies in the left ear. During the scanning session, subjects were also asked if they could clearly hear the sounds. Two subjects were excluded from data analysis because they indicated significant difficulty hearing the sounds.

### Stimuli and Task

Subjects completed an affective sound categorization task while in the fMRI scanner. Sounds from the International Affective Digital Sounds Database (IADS) with normative scores on valance (1 very unpleasant– 9 very pleasant) and arousal (1 low arousing– 9 high arousing) were used [39]. Thirty pleasant sounds (valance: 6.83 ± 0.54 arousal: 6.46 ± 0.56), 30 unpleasant sounds (valance: 2.78 ± 0.58 arousal: 6.9 ± 0.56) and, to serve as baseline in data analysis, 30 neutral sounds (valance: 4.81 ± 0.43 arousal: 4.85 ± 0.57) were presented to subjects during periods of “relative quiet” in the fMRI scanner in a sparse sampling paradigm [39]. Each participant was exposed to the same sounds for consistency. Sounds were presented at a most comfortable level during “relatively-quiet” time periods of clustered fMRI acquisition; this level ranged from 65–75 dB SPL. Sound delivery to the subjects was controlled using Presentation version 14.7 software (http://www.neurobs.com) on a Windows 7 machine. The Avotec silent scan 3300 sound system (http://www.avotecinc.com) was used to deliver sounds to the subjects through sound dampening headphones. Subjects were asked to rate the sounds as pleasant (P), unpleasant (U), or neutral (N) as soon as they felt confident in their rating using right hand button presses. Rating and reaction time data were collected.

### Data Acquisition

Functional and structural data were collected using a Magnetom Trio 3T fMRI scanner. Cluster echo-planar imaging (EPI) acquisition was used to reduce scanner noise interference with stimuli presentation [13, 40–43]. During a 9s interval, a 7s period of silence was used to present a 6s sound stimulus. A 2s scan followed the 7s interval of silence. Ninety functional images were collected in total. The following parameters were used to obtain the functional images: TR, 9000 ms with 2000 ms acquisition time; TE, 25 ms; slice thickness, 3 mm; inter-slice gap, 0.4 mm; 38 transverse slices, distance factor 10%; voxel size, 2.5 × 2.5 × 3.0 mm^3^. A low-resolution T2-weighted image (AxT2) was obtained using the following parameters: 38 low-resolution transversal slices (AxT2) (TR = 3400 ms, TE = 64.0 ms) with a 3.0 mm slice thickness and a 1.2 × 1.2 × 3.0 mm^3^ voxel size. A high resolution magnetization-prepared rapid-acquisition with gradient echo (MPRAGE) image was obtained using the following parameters: 160 MPRAGE sagittal slices that were 1.2 mm in thickness with a 1.0 × 1.0 × 1.2 mm^3^ voxel size (TR, 2300 ms; TE, 2.84 ms).

### Data Analysis

Behavioral data were analyzed using Statistical Package for Social Sciences version 22 software (SPSS, IBM,http://www-01.ibm.com/software/analytics/spss/). Group comparisons of age, tinnitus severity, physical activity level, hearing loss, anxiety and depression were computed using independent sample *t*-tests. Behavioral data from the scanning sessions were analyzed using ANOVA testing. For ANOVA testing, reaction time and ratings were set as dependent variables, and group (LD, HD) and condition (P, N, U) were set as independent variables. The statistical significance threshold was set at p<0.05.

Functional MRI data was analyzed using SPM8 software (Statistical Parametric Mapping, Welcome Trust Center for Neuroimaging, http://www.fil.ion.ucl.ac.uk/spm/software/spm8/) similar to our past studies [13, 42]. Images were preprocessed using the following steps: realignment, coregistration, normalization and smoothing (Gaussian kernel of 8 x 8 x 8mm^3^ FWHM) [13, 42]. In first level analysis, P>N and U>N contrast images were generated from each subject [13, 42]. At the second level, within a flexible factorial model, the P>N and U>N contrasts were combined into an Emotion>Neutral contrast in a manner similar to previous research in our lab [42]. Main effect of group and condition were carried out within the model, and whole brain post hoc independent sample *t*-tests were conducted to compare groups (i.e. HD>LD (Emotion>Neutral)). In addition to whole brain comparisons, we employed a region-of-interest (ROI) analysis based on our *a priori* hypothesis concerning the influence of the limbic system in processing sounds in individuals with tinnitus. This was similar to the analysis used in our previous study [13]. Anatomically defined regions of interested, including the insula, parahippocampus, and amygdala along with the primary auditory cortex (Brodmann areas 42, 41, 22), were generated using the Wake Forest University Pickatlas toolbox (http://www.fmri.wfubmc.edu). The auditory regions were included because tinnitus is an auditory disorder that has been shown to involve the auditory cortex [44–48]. For subsequent exploratory analysis, the LD and HD groups were divided into subgroups based on a median split of GTLEQ scores to investigate within group differences, henceforth referred to as the subgroup analyses. A full factorial ANOVA was conducted on the four subgroups (LD_HPS, LD_LPS; HD_HPS, HD_LPS) within SPM8 using distress and physical activity as factors. Subsequent within group independent sample *t*-tests comparing the subgroups using an ROI analysis comprised of the amygdala, parahippocampus, middle frontal gyrus, and superior frontal gyrus were conducted to further investigate the results of the between group comparisons (LD, HD). Statistical significance was set at p<0.025 FWE corrected for multiple comparisons for fMRI data analysis, and small volume correction (SVC) was applied to the ROI analysis. For the sub-group analyses, given the smaller group sizes and exploratory nature of the analysis investigating the effects of physical activity, we used a threshold of p<0.001 uncorrected.

## Results

### Demographic Results

Individuals were grouped based on tinnitus distress level and a group comparison of GTLEQ scores indicated groups also significantly differed in physical activity level. Groups did not significantly differ in age (p<0.219). A significant difference in THI and TFI score was observed between the groups, with the HD group scoring significantly higher on THI (34.5 ± 9.7; p<5.5x10^-9^) and TFI (46.1 ± 12.1; p<9.8x10^-10^) measures compared to the LD group (THI: 9.6 ± 5.7; TFI: 14.9 ± 7.6) (Table 1). The higher tinnitus distress group had significantly lower GLTEQ scores (27.3 ± 17.9; p<0.002) compared to the lower tinnitus distress group (57.7 ± 30.2) (Table 1). The HD group scored significantly higher on the BAI (4.9 ± 3.4; p<0.002) but not the BDI questionnaires (7.3 ± 7.5; p<0.087) compared to the LD group (BAI: 1.5 ± 2.1; BDI-II: 3.3 ± 4.6) (Table 1). Note that both groups scored within the same clinical category (minimal) for anxiety and depression. Note that those in the LD group had significantly higher levels of physical activity than the HD group. Correlational analysis between THI, TFI, GLTEQ, BAI and BDI-II was also conducted for supplementary information (S1 Table).

The same comparisons were computed within the HD subgroups and LD subgroups. The HD subgroups significantly differed in the GLTEQ (p<0.001) measure (Table 2). There was not a significant difference in age (p<0.192), THI (p<0.923), TFI (p<0.645), BAI (p<0.045) and BDI-II (p<0.658) (Table 2). Concerning the LD subgroups, a significant difference was observed in GLTEQ (p<0.001) (Table 2). There was not a significant difference in age (p<0.178), THI (p<0.425), TFI (p<0.594), BAI (p<0.013), or BDI-II (p<0.05)

### Behavioral Results

**Fig 2:**
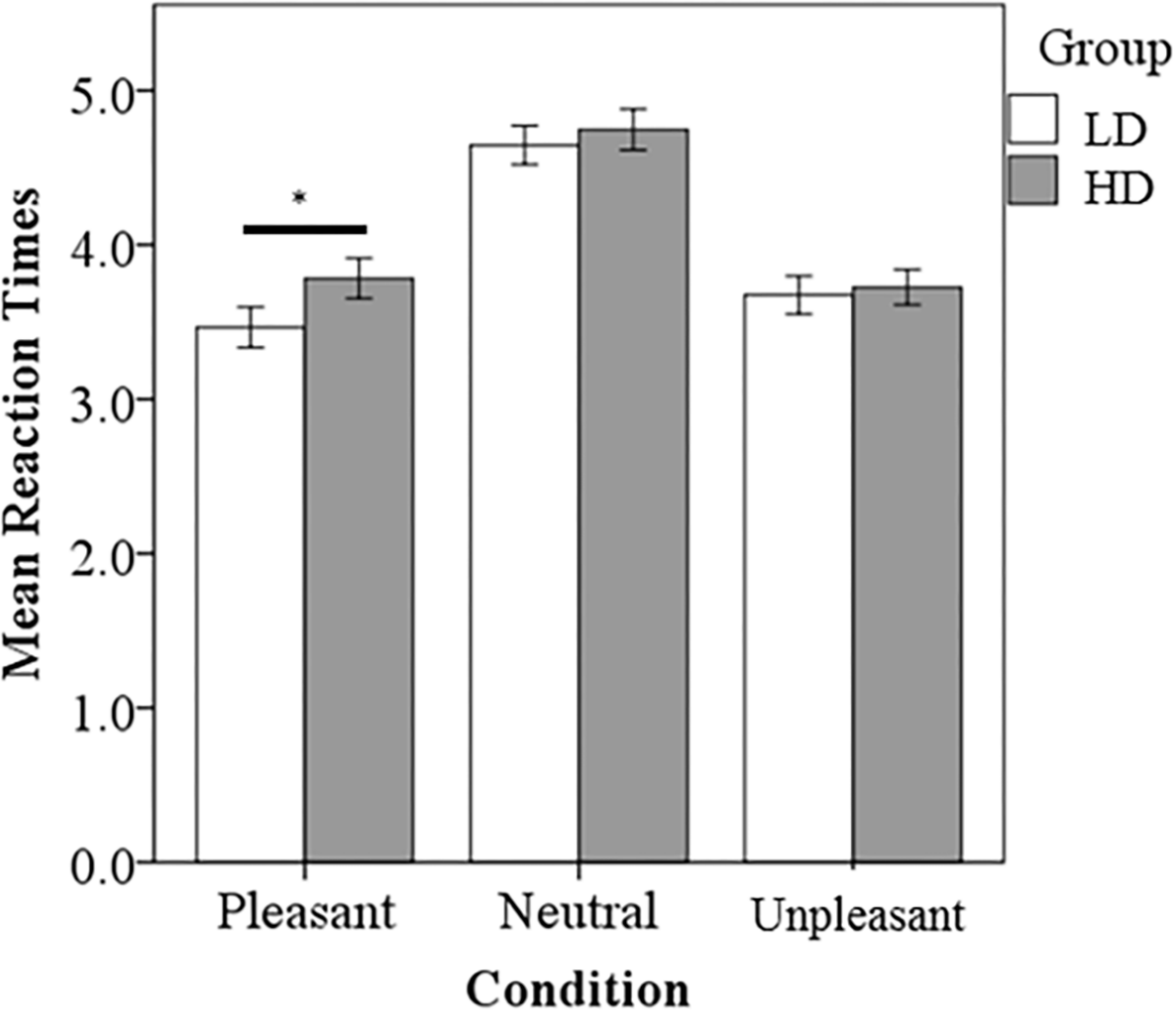
Task based reaction time results. The LD group responded significantly faster to P sounds compared to the HD group. Both groups responded significantly slower to N sounds compared to P and U sounds. Statistical significance level p<0.05 indicated by *.

**Fig 3:**
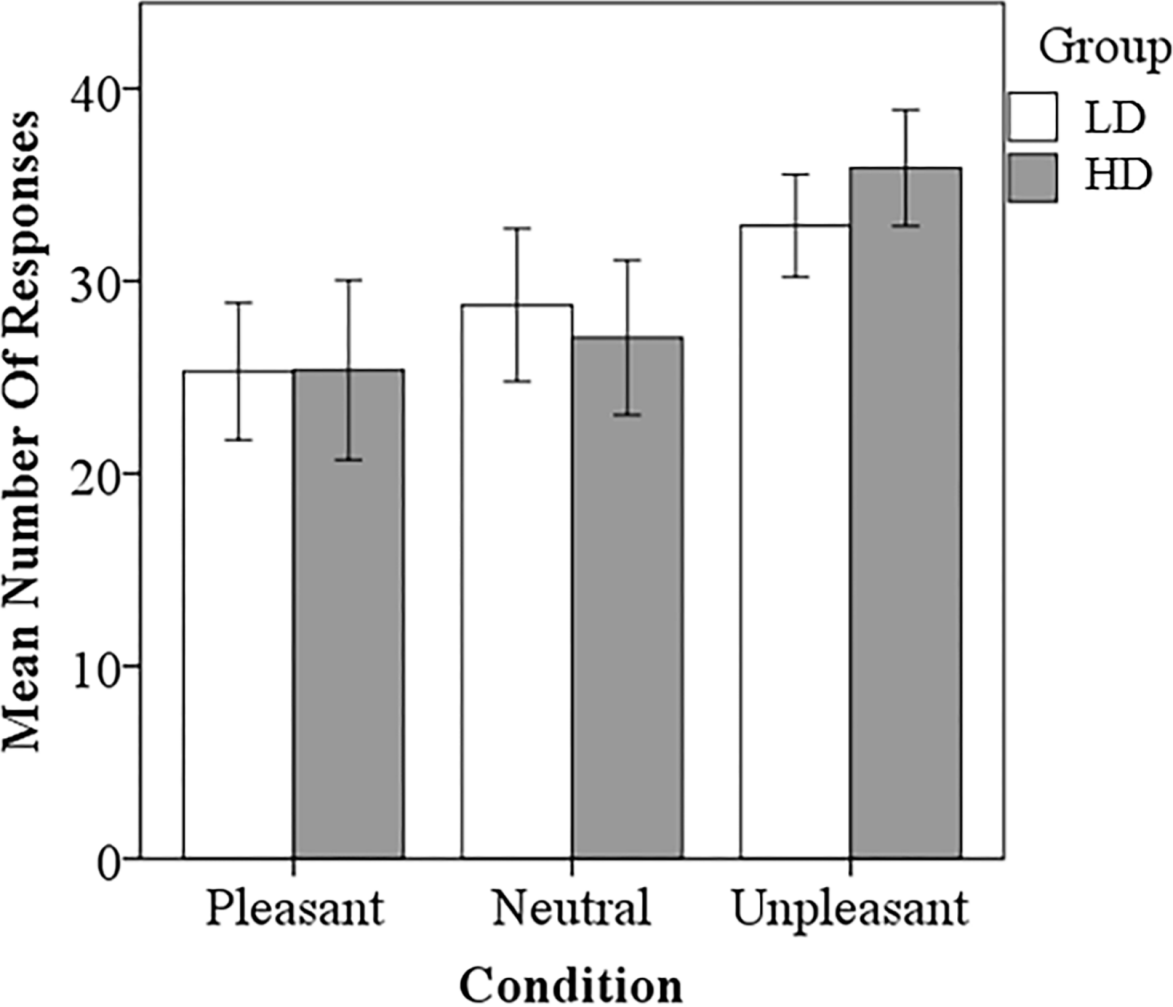
Task based rating results. No significant differences between the LD and HD group were detected. The LD group responded U significantly more than P. The HD group responded significantly more U than P and N. Statistical significance level p<0.05.

ANOVA testing was conducted to compare reaction time and responses. The LD group responded significantly faster to the pleasant sounds compared to the HD group (p<0.001) (Fig 2). There also appeared to be a trend for the LD group to respond faster to neutral and unpleasant sounds compared to the HD group, but this was not statistically significant. Both groups responded significantly slower to neutral sounds compared to pleasant (LD, p<0.001; HD, p<0.001) and unpleasant sounds (LD, p<0.001; HD, p<0.001), consistent with previous research [13]. The LD group responded significantly faster to pleasant sounds compared to the unpleasant sounds (p<0.020). Concerning the number of responses, no significant differences between groups were detected (Fig 3). The HD group responded unpleasant significantly more than pleasant (p<0.0001) and neutral (p<0.001). The LD group responded unpleasant significantly more than pleasant (p<0.005).

### fMRI Results

Main effect of group and condition were computed within the flexible factorial model using whole brain voxel-wise analysis. For main effect of group, neural responses were observed in the bilateral precentral gyrus, bilateral parahippocampus, right superior frontal gyrus, right inferior frontal gyrus, right amygdala, right inferior parietal lobule, left insula, left supramarginal gyrus, left superior temporal gyrus, left transverse temporal gyrus, left inferior parietal lobule, left precuneus, and cingulate gyrus (Table 3). For the main effect of condition, neural response was observed in the anterior cingulate (Table 3). Concerning the full factorial subgroup analysis, response was observed for main effect of distress, main effect of activity, and the interaction. The results from this analysis are detailed in Table 4. Note that the results did not change despite the inclusion of gender as a covariate during analysis.

**Table 3:** Local maxima for the main effect of group and condition.

Contrast	MNI Coordinates X, Y, Z	Z Score	Cluster Size (voxels)	Brain Region (Brodmann area)
**Main Effect of Group**	-24–24–26	6.42	73	L. Parahippocampus
	-28–52 38	5.77	342	L. Inferior Parietal Lobule (BA 40)
	-8–52 44	5.38		L. Precuneus (BA 7)
	32 4–28	6.00	102	R. Insula (BA 13
	-38 4 6	5.85	24	L. Insula (BA 13)
	30–14 60	5.75	85	R. Precentral Gyrus (BA 6)
	-22 4–20	5.56	24	L. Parahippocampus
	26 32 34	5.49	31	R. Superior Frontal Gyrus (BA 9)
	-6–2 42	5.48	31	Cingulate Gyrus (BA 24)
	-34 10–34	5.39	24	L. Superior Temporal Gyrus (BA 38)
	-60–48 30	5.38	26	L. Supramarginal Gyrus (BA 40)
	-58–18 12	5.38	32	L. Transverse Temporal Gyrus (BA 41)
	26–22–24	5.34	133	R. Parahippocampus
	22–4–22	5.69		R. Amygdala
	20–14–24	5.11		R. Parahippocampus
	-58–42 10	5.30	21	L. Superior Temporal Gyrus (BA 22)
	-52–48 14	5.16		L. Superior Temporal Gyrus (BA 22)
	32 42 26	5.26	12	R. Middle Frontal Gyrus (BA 10)
	54 18 24	5.23	17	R. Inferior Frontal Gyrus (BA 45)
	60–14 30	5.22	10	R. Precentral Gyrus (BA 4)
	-56–8 32	5.22	11	L. Precentral Gyrus (BA 6)
**Main Effect of Condition**	-4 40–4	5.59	1886	Anterior Cingulate (BA 32)

Regions are listed in Montreal Neurological Institute (MNI) coordinates. Brodmann areas are also provided (before determining the Brodmann areas, the MNI coordinates were converted to Talairach coordinates). Statistical threshold was set at p<0.025 FWE corrected for multiple comparisons. L, left; R, right.

**Table 4:** Subgroup analysis: local maxima for the main effect of distress and activity.

Contrast	MNI Coordinates X, Y, Z	Z Score	Cluster Size (voxels)	Brain Region (Brodmann area)
**Main Effect of Distress**	-28–32–28	4.45	158	L. Parahippocampus
	-24–24–26	3.32		L. Parahippocampus
	34 0–26	3.58	136	R. Parahippocampus
	30–22–24	3.48	236	R. Parahippocampus
	20–6–24	3.04		R. Amygdala
	28 38 38	3.48	167	R. Superior Frontal Gyrus (BA 9)
	-20–48 32	3.31	220	Cingulate Gyrus (BA 31)
	30–14 62	3.21	236	R. Precentral Gyrus (BA 6)
	-60–22–26	3.12	70	L. Inferior Temporal Gyrus (BA 20)
	54 20 18	3.08	66	R. Inferior Frontal Gyrus (BA 45)
**Main Effect of Activity**	-34–40–8	4.07	436	L. Parahippocampus
	-18–28–14	3.43		L. Parahippocampus
	-20–36–10	3.24		L. Parahippocampus
	24–8 28	3.69	111	Cingulate Gyrus
	44–18 22	3.56	81	R. Insula (BA 13)
	16 30 48	3.28	75	R. Superior Frontal Gyrus (BA 8)
	40–48 6	3.27	116	R. Middle Temporal Gyrus (BA 39)
	40–66 8	3.08		R. Middle Temporal Gyrus (BA 39)
**Interaction**	-50–40 24	4.09	146	L. Inferior Parietal Lobule (BA 40)
	-58–42 8	3.67	214	L. Superior Temporal Gyrus (BA 22)
	-52–40 2	3.36		L. Middle Temporal Gyrus (BA 22)
	-32 26 14	3.43	35	L. Insula (BA 13)
	62–34 8	3.35	91	R. Superior Temporal Gyrus (BA 22)
	-20 36 18	3.21	38	L. Medial Frontal Gyrus (BA 9)
	44–50 6	3.15	26	R. Middle Temporal Gyrus (BA 21)
	2 32 4	3.05	31	Anterior Cingulate (BA 24)
	-52 16 16	2.99	83	L. Inferior Frontal Gyrus (BA 44)

Regions are listed in Montreal Neurological Institute (MNI) coordinates. Brodmann areas are also provided (before determining the Brodmann areas, the MNI coordinates were converted to Talairach coordinates). Statistical threshold was set at p<0.001 uncorrected. L, left; R, right.

Within group whole brain voxel-wise analysis revealed elevated response in temporal regions in response to the affective stimuli for both groups compared to neutral sounds. For the HD (Emotion>Neutral) contrast, elevated response was observed in bilateral superior temporal gyrus, right middle temporal gyrus, bilateral amygdala, and bilateral parahippocampus (Table 5). For the LD (Emotion>Neutral) contrast, heightened response was observed in the bilateral superior temporal gyrus, left middle temporal gyrus, left superior frontal gyrus and medial frontal gyrus (Table 5). Note that increased amygdala and parahippocampal activity was not detected for the LD (Emotion>Neutral) contrast (Table 5). This is consistent with past research in our lab, which detected heightened temporal response in tinnitus groups to affective stimuli [13]. Note that in the previous studies, the tinnitus groups had mild tinnitus. For a complete list of activated regions, refer to Table 5.

**Table 5:** Local maxima for the whole-brain analysis for within group contrasts.

Contrast	MNI Coordinates X, Y, Z	Z Score	Cluster Size (voxels)	Gyrus (Brodmann area)
**HD Emotion>Neutral**	56–4 2	8.11	2510	R. Superior Temporal Gyrus (BA 22)
	60–16–14	6.22		R. Middle Temporal Gyrus (BA 21)
	66–28–4	6.29		R. Middle Temporal Gyrus (BA 21)
	-60–28 6	7.70	2558	L. Superior Temporal Gyrus (BA 22)
	-54–16–2	7.67		L. Superior Temporal Gyrus (BA 22)
	-56–34 14	7.48		L. Superior Temporal Gyrus (BA 42)
	22–4–20	7.68	542	R. Amygdala
	26 2–16	7.64		R. Parahippocampus
	-22 2–18	7.57	771	L. Amygdala
	-38 0–22	6.53		L. Parahippocampus
	-2 54 22	7.50	898	Medial Frontal Gyrus (BA 10)
	-8 54 14	7.00		Medial Frontal Gyrus (BA 9)
	-8 44 14	6.90		Medial Frontal Gyrus (BA 10)
	-24–24–26	6.55	111	L. Parahippocampus
**LD Emotion>Neutral**	58 0 0	8.55	1407	R. Superior Temporal Gyrus (BA 22)
	60–16 6	7.70		R. Superior Temporal Gyrus (BA 22)
	64–26 0	7.48		R. Inferior Frontal Gyrus (BA 47)
	-60–22 6	8.30	3288	L. Superior Temporal Gyrus (BA 22)
	-52–24 14	7.55		L. Superior Temporal Gyrus (BA 22)
	-62–20–6	7.50		L. Middle Temporal Gyrus (BA 21)
	-10 54 14	7.71	871	Medial Frontal Gyrus (BA 10)
	-14 50 36	6.58		L. Superior Frontal Gyrus (BA 10)
	-6 46 28	6.50		Medial Frontal Gyrus (BA 10)
	-6–2 40	6.58	224	L. Cingulate Gyrus (BA 24)
	-26–42 72	6.39	137	L. Postcentral Gyrus (BA 7)
	-8–54 42	6.13	176	L. Precuneus (BA 7)
	-34 18–14	6.09	55	L. Inferior Frontal Gyrus (BA 47)
	-4 34–6	6.07	361	Anterior Cingulate (BA 32)
	-2 52–12	5.95		Medial Frontal Gyrus (BA 11)
	10 54–10	5.86		Medial Frontal Gyrus (BA 11)

Whole-brain analysis for both the P>N and U>N conditions was computed for each group. Regions are listed in Montreal Neurological Institute (MNI) coordinates. Brodmann areas are also provided (before determining the Brodmann areas, the MNI coordinates were converted to Talairach coordinates). Statistical threshold was set at p<0.025 FWE corrected for multiple comparisons. L, left; R, right.

#### Heightened amygdala and parahippocampal response in HD compared to LD

**Fig 4:**
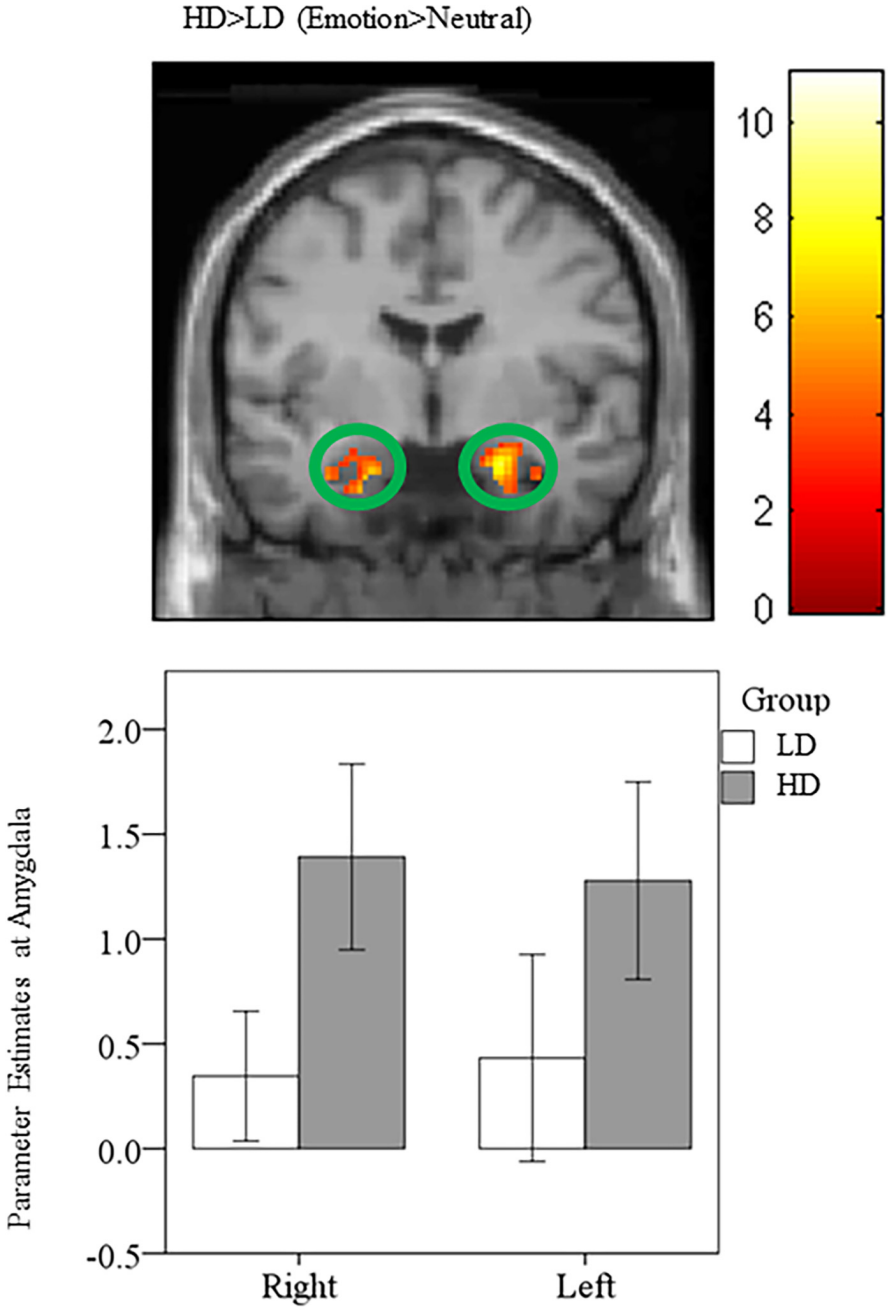
Statistical parametric maps for amygdala region-of-interest (ROI). Post-hoc independent sample t-tests using ROI analysis revealed heightened response in the bilateral amygdala for the HD>LD (Emotion>Neutral) comparison. For illustration purposes, the ROI for the amygdala is shown at p<0.001 uncorrected, but the clusters in the circles are corrected for multiple comparisons (p<0.025 FWE).

Groups were compared using post-hoc independent sample *t*-tests. Concerning whole brain analysis, increased response was observed in bilateral amygdala and bilateral parahippocampus for the HD>LD (Emotion>Neutral) comparison (Table 6). This is consistent with previous research in our lab that showed similar increases in parahippocampal response in mild tinnitus groups compared to non-tinnitus controls [13]. Similarly, ROI analysis of post-hoc independent sample *t*-tests detected elevated response in bilateral amygdala and bilateral parahippocampus for the HD>LD (Emotion>Neutral) contrast (Fig 4; Table 7).

**Table 6:** Whole Brain.

Contrast	MNI Coordinates X, Y, Z	Z Score	Cluster Size (voxels)	Gyrus (Brodmann area)
**HD>LD Emotion>Neutral**	26–22–24	6.91	225	R. Parahippocampus
	22–4–22	5.81		R. Amygdala
	20–14–24	5.24		R. Parahippocampus
	-24–24–26	6.53	125	R. Parahippocampus
	-20 4–22	5.68	42	L. Amygdala
**LD>HD Emotion>Neutral**	-38 4 6	5.96	32	L. Insula (BA 13)
	-38–12 60	5.87	121	L. Precentral Gyrus (BA 6)
	26 32 34	5.61	172	R. Superior Frontal Gyrus (BA 9)
	32 42 26	5.39		R. Middle Frontal Gyrus (BA 10)
	30 44 32	5.08		R. Superior Frontal Gyrus (BA 9)
	-22 54 26	5.33	145	L. Middle Frontal Gyrus (BA 10)
	-28 32 34	5.12		L. Superior Frontal Gyrus (BA 9)
	-60–48 30	6.41	45	L. Supramarginal Gyrus (BA 40)
	-46–52 32	5.58		L. Supramarginal Gyrus (BA 40)
	-58–18 12	5.50	45	L Transverse Temporal gyrus (BA 41)
	32 42 26	5.36	24	R. Middle Frontal Gyrus (BA 10)
	52 18 22	5.36	36	R. Inferior Frontal Gyrus (BA 45)

Local maxima for the whole-brain independent sample *t*-tests. Whole-brain independent sample *t*-tests were computed for between group differences. Regions are listed in Montreal Neurological Institute (MNI) coordinates. Brodmann areas are also provided (before determining the Brodmann areas, the MNI coordinates were converted to Talairach coordinates). Statistical threshold was set at p<0.025 FWE corrected for multiple comparisons. L, left; R, right.

**Table 7:** ROI.

Contrast	MNI Coordinates X, Y, Z	Z Score	Cluster Size (voxels)	Gyrus (Brodmann area)
**HD>LD Emotion>Neutral**	26–22–24	6.91	617	R. Parahippocampus
	22–4–22	5.81		R. Amygdala
	34 2–26	5.33		R. Parahippocampus
	-24–24–26	6.53	557	L. Parahippocampus
	-22 4–20	5.68		L. Amygdala
	-32–12–32	5.11		L. Parahippocampus
**LD>HD Emotion>Neutral**	-38 2 8	5.96	116	L. Insula (BA 13)
	-58–18 12	5.50	96	L Transverse Temporal gyrus (BA 41)
	62–16 10	5.06	96	L Transverse Temporal gyrus (BA 42)

Local maxima for the region of interest (ROI) independent sample *t*-tests. Between group independent sample *t-*tests using ROI analysis comprised of amygdala, insula, parahippocampus, and primary auditory cortex (Brodmann areas 42, 41, 22) was conducted. Regions are listed in Montreal Neurological Institute (MNI) coordinates. Brodmann areas are also provided (before determining the Brodmann areas, the MNI coordinates were converted to Talairach coordinates). Statistical threshold was set at p<0.025 FWE corrected for multiple comparisons. L, left; R, right.

**Fig 5:**
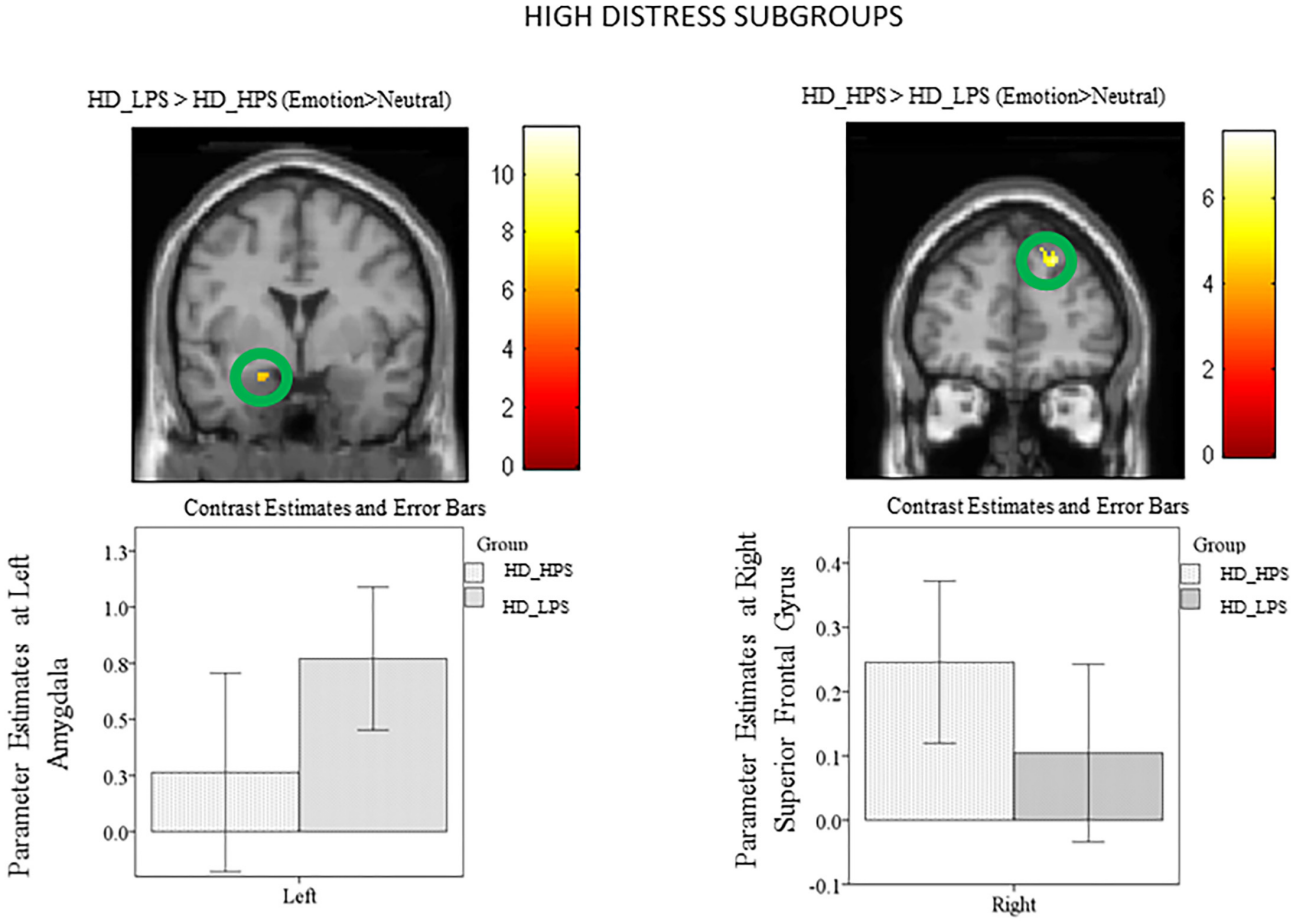
Statistical parametric maps for amygdala region-of-interest (ROI). Post-hoc independent sample t-tests using ROI analysis revealed heightened response in the left amygdala for the HD_LPS > HD_HPS (Emotion>Neutral) comparison and increased response in the right superior frontal gyrus for the HD_HPS > HD_LPS (Emotion>Neutral) comparison. The clusters in the circles are significant at p<0.001 uncorrected.

**Fig 6:**
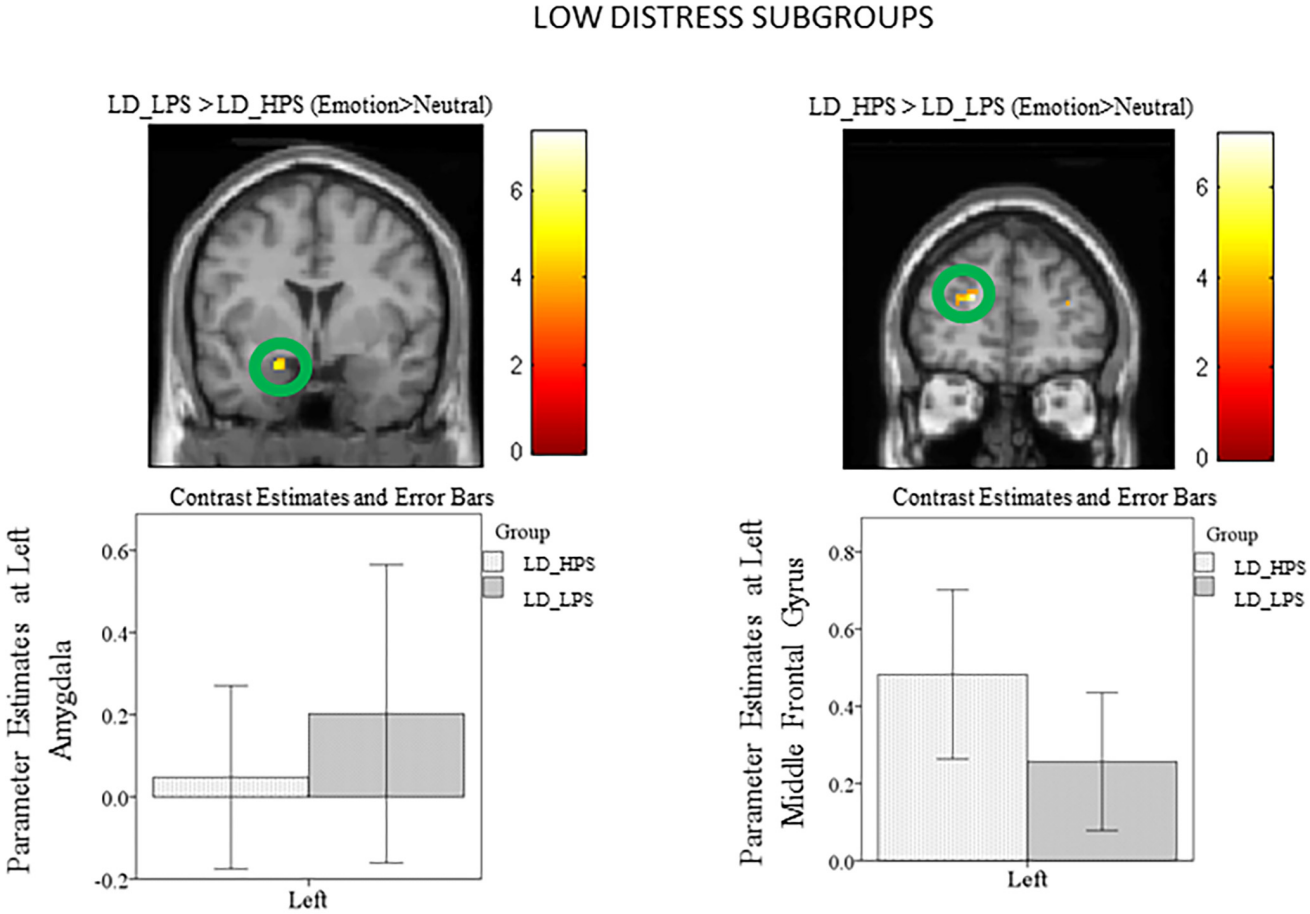
Statistical parametric maps for amygdala region-of-interest (ROI). Post-hoc independent sample t-tests using ROI analysis revealed heightened response in the left amygdala for the LD_LPS > LD_HPS (Emotion>Neutral) comparison and increased response in the left middle frontal gyrus for the reverse comparison. The clusters in the circles are significant at p<0.001 uncorrected.

Subgroups: ROI analysis using post-hoc independent sample *t*-tests of the subgroups revealed increased left parahippocampus and left amygdala activity in the HD_LPS> HD_HPS (Emotion>Neutral) comparison (Fig 5; Table 8). Similarly, heightened response was observed in the bilateral parahippocampus and the left amygdala for the LD_LPS> LD_HPS (Emotion>Neutral) contrast (Fig 6; Table 9). Limbic response was not observed in the reverse comparisons (Table 9).

**Table 8:** HD Subgroup.

Contrast	MNI Coordinates X, Y, Z	Z Score	Cluster Size (voxels)	Gyrus (Brodmann area)
**HD_LPS> HD_HPS Emotion>Neutral**	-28–50–6	5.66	559	L. Parahippocampus
	-26–6–14	5.03	15	L. Amygdala
**HD_HPS> HD_LPS Emotion>Neutral**	20 44 38	3.74	24	R. Superior Frontal Gyrus (BA 9)
	14 44 44	3.39		R. Superior Frontal Gyrus (BA 8)
	20 32 46	3.70	51	R. Middle Frontal Gyrus (BA 8)

Local maxima for the region of interest (ROI) independent sample *t*-tests for the within group split of the HD group based on median GTLEQ score. ROI analysis, comprised of amygdala, insula, parahippocampus, superior frontal gyrus, middle frontal gyrus, inferior frontal gyrus, and auditory cortex (Brodmann areas 42, 41, 22), was conducted. Regions are listed in Montreal Neurological Institute (MNI) coordinates. Brodmann areas are also provided (before determining the Brodmann areas, the MNI coordinates were converted to Talairach coordinates). Statistical threshold was set at p<0.001 uncorrected. L, left; R, right.

**Table 9:** LD Subgroup.

Contrast	MNI Coordinates X, Y, Z	Z Score	Cluster Size (voxels)	Gyrus (Brodmann area)
**LD_LPS> LD_HPS Emotion>Neutral**	34–20–18	4.64	80	R. Parahippocampus
	-20–32–12	4.02	98	L. Parahippocampus
	-22 2–18	3.73	18	L. Amygdala
**LD_HPS> LD_LPS Emotion>Neutral**	-20 46 20	4.58	47	L. Superior Frontal Gyrus (BA 10)
	28–18 50	4.35	51	R. Middle Frontal Gyrus (BA 6)
	26 24 44	3.96	27	R. Middle Frontal Gyrus (BA 8)
	52 4 44	3.32	22	R. Middle Frontal Gyrus (BA 8)

Local maxima for the region of interest (ROI) independent sample *t*-tests for the within group split of the LD group based on median GTLEQ score. ROI analysis, comprised of amygdala, insula, parahippocampus, superior frontal gyrus, middle frontal gyrus, inferior frontal gyrus, and auditory cortex (Brodmann areas 42, 41, 22), was conducted. Regions are listed in Montreal Neurological Institute (MNI) coordinates. Brodmann areas are also provided (before determining the Brodmann areas, the MNI coordinates were converted to Talairach coordinates). Statistical threshold was set at p<0.001 uncorrected. L, left; R, right.

#### Elevated frontal response in LD compared to HD

**Fig 7:**
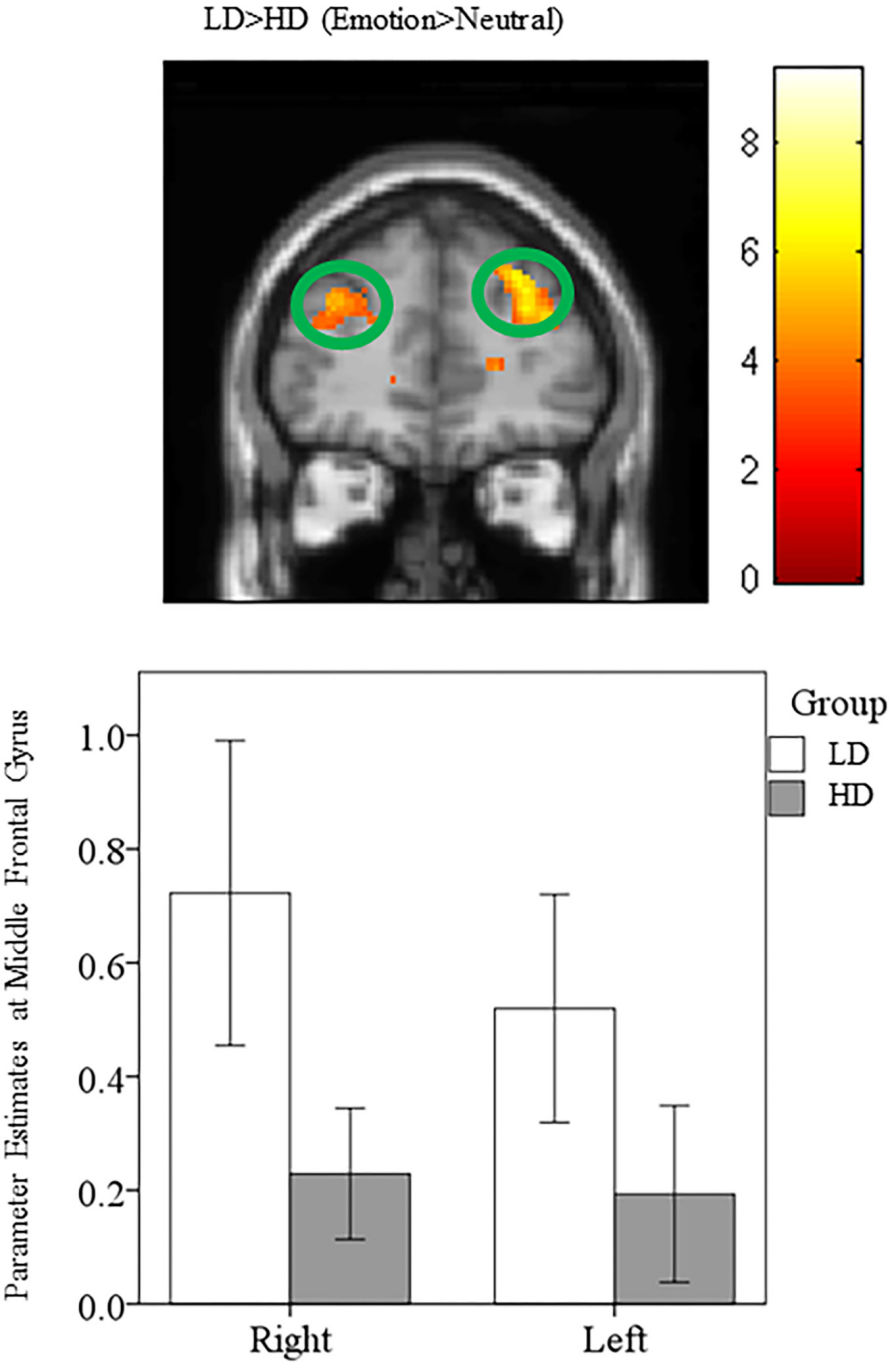
Statistical parametric maps for middle frontal gyrus. Post-hoc independent sample t-tests using whole brain analysis revealed heightened response in the bilateral middle frontal gyrus for the LD>HD (Emotion>Neutral) comparison. For illustration purposes the comparison is displayed at p<0.001 uncorrected, but the clusters in the circles are corrected for multiple comparisons (p<0.025 FWE). Note the peak voxel for the left middle frontal gyrus is slightly posterior to the circled area of activation.

For whole brain post-hoc independent sample *t*-tests, elevated response was observed in the bilateral superior frontal gyrus, bilateral middle frontal gyrus, and right inferior frontal gyrus for the LD>HD (Emotion>Neutral) comparison (Fig 7, Table 6). For the ROI analysis of the post-hoc independent sample *t*-tests, heightened response was observed in the left transverse temporal gyrus and left insula for the LD>HD (Emotion>Neutral) comparison (Table 7). For a complete list of activated regions, refer to Tables 4 and 5.

Subgroups: For the ROI analysis of the post-hoc independent sample *t*-tests, increased response in the right superior frontal gyrus and the right middle frontal gyrus was observed in the HD_HPS> HD_LPS (Emotion>Neutral) comparison (Fig 6, Table 8). Likewise, heightened response was observed in the left superior frontal gyrus and the right middle frontal gyrus for the LD_HPS> LD_LPS (Emotion>Neutral) contrast (Fig 6, Table 9). Frontal response was not observed in the reverse comparisons (Table 9).

We also performed a subgroup analysis to compare the HD_HPS and LD_LPS groups. These groups do not differ in GTLEQ, BAI, or BDI scores, but did differ significantly on both measures of tinnitus severity (THI and TFI p<0.001). This allowed us to examine the effects of tinnitus severity independent of physical activity, depression and anxiety. Only one significant difference was found; increased response in the right middle occipital gyrus in the LD_LPS>HD_HPS (Emotion>Neutral) comparison (Table 10). No suprathreshold voxels were evident in the ROI analysis of these subgroups.

**Table 10:** Local maxima for the whole-brain independent sample *t*-tests contrasting the LD_LPS and HD_HPS groups.

Contrast	MNI Coordinates X, Y, Z	Z Score	Cluster Size (voxels)	Gyrus (Brodmann area)
**HD_HPS>LD_LPS Emotion>Neutral**				No Suprathreshold voxels
**LD_LPS>HD_HPS Emotion>Neutral**	44–60 4	3.99	14	R. Middle Occipital Gyrus (BA19)

Whole-brain independent sample *t*-tests were computed for between group differences. Regions are listed in Montreal Neurological Institute (MNI) coordinates. Brodmann areas are also provided (before determining the Brodmann areas, the MNI coordinates were converted to Talairach coordinates). Statistical threshold was set at p<0.001 uncorrected. L, left; R, right.

## Discussion

Our results yielded two main findings: (1) Increased response in the amygdala and parahippocampus was observed in the HD group compared to the LD group, and (2) The LD group had increased frontal response compared to the HD group. Additionally, our results from the secondary exploratory analysis suggested that lower levels of physical activity may be associated with increased response in the limbic system, and that higher physical activity levels may contribute to heightened response in frontal regions. These findings are consistent with our initial hypotheses and are discussed in turn below.

### Heightened amygdala and parahippocampal response in HD compared to LD.

Based on the literature, we expected the HD group to show increased engagement of the amygdala and parahippocampus compared to the LD group [12, 13, 15, 49–51]. Our results supported our hypothesis, and increased amygdala and parahippocampal response was observed in the HD>LD (Emotion>Neutral) contrast. This is corroborated by previous research that used low resolution electromagnetic tomography to investigate neural correlates of tinnitus distress and found increased activation of the amygdala to be correlated with tinnitus distress [12]. Past research using the same experimental paradigm as the present study substantiates these findings [13]. In our previous study, we found that individuals with lower levels of tinnitus severity relied less on the amygdala when processing affective sounds than normal hearing and hearing loss control groups [13]. We surmised that the lower severity tinnitus group relied on alternative regions of the limbic system to avoid the amygdala and its connections with the auditory cortex to minimize the bothersome nature of tinnitus, and additionally, that those with higher levels of tinnitus severity would recruit the amygdala to a greater extent [13, 16]. In addition, heightened parahippocampal response in the HD group compared to the LD group when listening to affective stimuli may be an indication of tinnitus distress. In support of this hypothesis, past research found increased parahippocampal response in tinnitus compared to non-tinnitus groups [13]. Other studies have also shown increased parahippocampal response to be associated with tinnitus distress [11]. Increased parahippocampal response has been associated with the lack of habituation to novel stimuli and tinnitus-related distress [12].

The subgroup analysis revealed that individuals with lower levels of physical activity, in both higher and lower tinnitus distress groups, recruited the amygdala and parahippocampus to a greater extent than those with higher levels of physical activity; note that these differences were significant at a threshold of p<0.001 uncorrected. We suspect the elevated limbic response in the lower physical activity level subgroups compared to higher physical activity subgroups may reflect a diminished capacity to engage frontal regions of the brain to regulate the emotional reaction to tinnitus. The impact of the engagement of frontal regions has been noted in our previous studies [13, 14] and is discussed further in the next section of this paper. Therefore, increased response from amygdala and parahippocampal regions may be an indication of tinnitus related distress, and lower physical activity levels may be associated with the observed increased recruitment of the limbic system in the higher tinnitus distress group.

### Elevated frontal response in LD compared to HD

Consistent with our hypothesis, increased response in frontal regions was detected in the LD group compared to the HD group when processing affective sounds. We propose that the observed increased frontal response in the LD group is an indication of successful habituation to the tinnitus percept through improved regulation of emotional response. This is supported by previous work that demonstrated that increased frontal response was associated with top down control over emotional processing [19, 52]. A recent review suggested that the use of top down mechanisms to exert control over emotional reactions can, over time, lead to the ability to cope with emotional distress [19]. Similar results were also observed when comparing mild tinnitus to normal hearing controls in our previous study (13), suggesting that increased frontal response may be necessary to reduce tinnitus distress. In contrast to our findings, a recent EEG study suggested maladaptive coping may be related to increased alpha activity in the frontal cortex [53]. However, we suspect that the differences between those results and our own may be attributed to the task, in that the EEG data was collected in a dimly lit room while the subjects were seated, as opposed to an affective sound categorization task used in the current study. Alternatively, in more severe forms of tinnitus (severe-catastrophic THI ratings) not included in the present study, increased frontal response may be an unsuccessful attempt to reduce the emotional reaction to tinnitus.

In the subgroup analysis, we found those with higher physical activity recruited more frontal regions while listening to emotional sounds when compared to those with lower levels of physical activity. Note that the subgroup differences were significant at p<0.001 uncorrected. Our results suggest successful coping to tinnitus may involve increased frontal response, and higher levels of physical activity may contribute to the observed increase in frontal response in the LD group compared to the HD group.

To further support this claim, we compared the HD_HPS and LD_LPS groups directly. These groups only differed in tinnitus severity measures and had similar levels of physical activity (see Table 2). This allowed us to investigate the effect of tinnitus severity alone on activity during the task. Only one significant result was found in the whole brain analysis; in the LD_LPS group compared to the HD_HPS group, increased response was noted in the right middle occipital gyrus (see Table 10). These results suggest that variables aside from tinnitus severity may be responsible for the differences observed between the two groups. Differences in physical activity may therefore be a strong contributor to the increased activity in frontal areas noted in the LD group compared to the HD group. The lack of differences in the response of the limbic areas between the groups may be due to a lack of differences in depression and anxiety between the HD_HPS and LD_HPS groups. However, there was also no categorical difference in BAI and BDI scores between the LD and HD groups, so this may not adequately explain the lack of limbic differences seen in this subgroup analysis. The relationship between tinnitus severity, physical activity, anxiety and depression warrants further investigation and is discussed further in the Caveats section of this paper.

Increased response in temporal regions may be associated with decreased tinnitus severity. Previous research has found increased recruitment of auditory regions in those that have had tinnitus for a long period of time compared to those that were recently developed chronic tinnitus [48]. The Vanneste et al. (2011) group suggested the observed increased response in the temporal cortex was due to tonal memory of the tinnitus percept [48]. Alternatively, we suggested increased temporal response may be associated with habituation to the tinnitus percept. In the current study, the increased temporal response in the lower distress group compared to the higher distress group when listening to affective sounds may be associated with successful habituation to tinnitus.

Contrary to our hypothesis, increased insular activation was observed in the LD group rather than the HD group. Consistent with our finding, a previous study using real time fMRI feedback asked participants with tinnitus to attempt to decrease their tinnitus [54]. They found that when subjects successfully decreased their tinnitus, they relied on increased insular activation [54]. Similarly, past research found increased insula activation in individuals with lower levels of tinnitus severity [13]. These discrepancies may be better explained by an analysis to identify the specific region of the insula involved, as recent work has suggested different areas of the insula may have varying functions [55]. In short, contrary to our hypothesis, our results suggest the increased insular activity may be associated with lower levels of tinnitus distress, and an analysis to identify the specific portion of the insula involved may help to clarify this finding.

## Caveats

Because neutral sounds were used as a baseline, functional differences in response to neutral sounds between groups were not discernable. The sounds were broad-band with varying peak and average amplitudes [39]. No attempt was made to control for the energy of the presented sounds during scanning, nor were the sounds altered to fit each patient’s hearing loss profile. Instead, we decided to preserve the ecological validity of the sounds and present them unaltered. Despite this, the average ratings of sounds did not differ between groups. We used the individual subject’s subjective ratings to classify each sound in accordance with previous studies from our lab [13, 14, 42] and elsewhere [56].

We asked participants if they were employing therapies for tinnitus, and while one volunteer indicated using the dietary supplement Lipoflavonoid in the past for his tinnitus, all other volunteers indicated they had not used any form of therapy. Therefore it is unlikely that any past tinnitus treatments impacted our results.

The cross sectional design of the study also precludes us from observing longitudinal changes in emotional processing that may occur from changing physical activity levels. Therefore, we can only speculate as to the involvement of physical activity in the habituation and reduced severity of tinnitus. Further investigation is warranted before substantial claims may be made concerning the benefits of physical activity on tinnitus distress; please refer to our recent survey paper (9) for additional results concerning this relationship. Additionally, it is unclear if the minimal differences in anxiety (BAI) and depression (BDI) were due to tinnitus distress or an unrelated issue. To address this, we divided subjects into two groups based on BAI and BDI scores along the median, instead of THI, resulting in high/low anxiety/depression groups instead of high/low severity groups, and found that this did not significantly affect group membership. These similar groupings support our assumption that BAI and BDI-II scores may be the result of tinnitus distress. But, we cannot sufficiently differentiate between the anxiety and depression caused by tinnitus and any pre-existing or non-tinnitus related levels. Further study of individuals before they develop tinnitus is necessary to distinguish pre-existing depression and anxiety from that attributable to tinnitus.

It is also worth noting that subjective loudness of tinnitus has been shown to be a component of tinnitus distress [57]. In this study, the metrics used to assess tinnitus distress (TFI and THI) include questions pertaining to subjective loudness of tinnitus (the louder the perceived tinnitus, the higher the score). Therefore, subjective loudness is combined with other metrics to determine tinnitus severity, and separating the effects of tinnitus loudness and severity is not possible. However, we also asked a stand-alone question about the perception of tinnitus loudness. The results from our subjective loudness scale (0: loudness rated extremely weak– 100: loudness rated extremely loud) showed no significant difference (p = .277) between the high (37 ± 15) and low (30 ± 17) severity groups. Three subjects from each group did not complete the subjective loudness assessment. Given that the two groups do not differ in terms of this measure, it is unlikely that subjective loudness is responsible for the differences in activity between the high and low severity groups. However, we cannot dissociate between subjective tinnitus loudness and the measures used to assess tinnitus severity. Despite this and the other confounds discussed, the present study serves as a foundation for a future pilot study to investigate neural changes that may occur after a physical activity intervention.

## Conclusion

We found evidence for the engagement of limbic and frontal regions in mediating tinnitus distress. We observed that individuals with higher levels of tinnitus distress recruited amygdala and parahippocampal gyrus to a larger extent than individuals with lower tinnitus distress. Increased frontal response was observed in the low distress group compared to the high distress group when listening to affective stimuli. Therefore, individuals that have lower levels of tinnitus distress may utilize frontal regions to control their emotional response to tinnitus and reduce tinnitus severity. These results were also observed in an exploratory subgroup analysis with groups stratified based on physical activity level, suggesting that physical activity may contribute to the observed results. Increased levels of physical activity may be associated with increased response from frontal regions and improved emotional regulation. Our study also serves as a baseline for assessing the efficacy of physical activity as a tinnitus treatments. We propose that limbic and frontal regions are key areas that should be examined in tinnitus intervention studies, and we recommend further investigation concerning the association between physical activity and tinnitus distress. We suspect increased limbic response will be observed pre-intervention and increased frontal response post-intervention with the use of any tinnitus intervention, such as physical activity.

## Supporting Information

S1 Table Pearson’s correlational analysis. THI = Tinnitus Handicap Inventory, TFI = Tinnitus Functional Index, GLTEQ = Godin leisure time exercise questionnaire, BAI = Beck anxiety inventory, BDI-II = Beck depression inventory.
(DOCX)

## Abbreviations

TFI: Tinnitus Functional Index
THI: Tinnitus Handicap Inventory
GTLEQ: Godin Leisure Time Exercise Questionnaire
BAI: Beck Anxiety Inventory
BDI-II: Beck Depression Inventory
HD: Higher Distress tinnitus
LD: Lower Distress tinnitus
HPS: Higher Physical Activity Level Subgroup
LPS: Lower Physical Activity Level Subgroup
