# Supplementary material for: Increased Frontal Response May Underlie Decreased Tinnitus Severity
Source: PLoS One. 2015 Dec 14;10(12):e0144419. doi: 10.1371/journal.pone.0144419 (PMC4684365; doi:10.1371/journal.pone.0144419)
Supplement: S1 Table — THI = Tinnitus Handicap Inventory, TFI = Tinnitus Functional Index, GLTEQ = Godin leisure time exercise questionnaire, BAI = Beck anxiety inventory, BDI-II = Beck depression inventory. (DOCX) [file pone.0144419.s001.docx]

**Supplementary Table 1.**

|  | **THI** | **TFI** | **BAI** | **BDI-II** |
| --- | --- | --- | --- | --- |
| **GLTEQ** Pearson Correlation  Sig. (2-tailed) | -.498  .004 | -.505  .003 | -.562  .001 | -.387  .029 |
| **BAI** Pearson Correlation  Sig. (2-tailed) | .523  .002 | .454  .009 |  | .686  <.0001 |
| **BDI -II** Pearson Correlation  Sig. (2-tailed) | .192  .294 | .183  .317 | .686  <.0001 |  |

**Supplementary Table 1.** Pearson’s correlational analysis. THI = Tinnitus Handicap Inventory, TFI = Tinnitus Functional Index, GLTEQ = Godin leisure time exercise questionnaire, BAI = Beck anxiety inventory, BDI-II = Beck depression inventory.
